# A Case of Syringocystadenocarcinoma Papilliferum: Not Your Usual Suspect

**DOI:** 10.7759/cureus.39424

**Published:** 2023-05-24

**Authors:** Alena Bashinskaya, Lucas Shapiro, Conner Thompson, Artis P Truett, Patricia Moody

**Affiliations:** 1 Osteopathic Medicine, Nova Southeastern University Dr. Kiran C. Patel College of Osteopathic Medicine, Fort Lauderdale, USA; 2 Dermatology, Owensboro Dermatology, Owensboro, USA; 3 Dermatopathology, KorPath, Tampa, USA

**Keywords:** syringocystadenoma papilliferum, nevus sebaceous, skin histopathology, adnexal skin tumor, scalp lesion, dermatology, syringocystadenocarcinoma papilliferum

## Abstract

Syringocystadenocarcinoma papilliferum (SCACP) is an exceptionally rare cutaneous adnexal tumor that is infrequently encountered by clinicians worldwide. The tumor typically appears in the older population, affecting patients in their fifth and sixth decades of life without male or female predominance. Patients frequently present with a variable-sized hyperpigmented ulcerative lesion containing an exudate that has a long-standing course of progression from its benign counterpart, Syringocystadenoma papilliferum (SCAP). Additionally, the clinical presentation and morphology of the neoplasm can be easily confused with a variety of other skin cancers, such as squamous cell carcinoma (SCC), basal cell carcinoma (BCC), cutaneous lymphoma, and cutaneous metastasis. Therefore, histopathology and tissue analysis play an essential role in establishing an accurate diagnosis. However, the lesion is so rare that no definitive diagnostic markers have been established yet. We present a case of SCACP localized to the scalp of the patient. Our case study highlights the presence of specific tumor markers that could potentially serve as objective criteria for diagnosis.

## Introduction

Cutaneous adnexal neoplasms are notorious for masquerading as skin cancers due to their close proximity to the skin; thus, they should be considered on the list of differential diagnoses for suspicious skin lesions [1]. The majority of adnexal neoplasms arise from apocrine and eccrine sweat glands due to an unknown etiology, but hypothetical causes include UV radiation, immunosuppression, trauma to the affected area, and genetic predisposition [1]. For instance, mutations in the epidermal growth factor receptor (EGFR), Wnt family member 10A (WNT10A), HRas proto-oncogene (HRAS), and retinoblastoma (RB1) genes have been associated with the tumorigenesis of benign and malignant adnexal neoplasms [1,2]. 

Syringocystadenocarcinoma papilliferum (SCACP) is a rare cutaneous adnexal neoplasm that typically arises from its benign precursor lesion, syringocystadenoma papilliferum (SCAP), or nevus sebaceous (NS) [3-5]. The tumor is described as a malignant proliferation of adnexal tissue arising in a raised nodule or an inflammatory plaque that develops from apocrine or pluripotent appendageal glands [3,4]. Human papillomavirus (HPV) has been reported to promote the malignant progression of SCAP into SCACP [6]. Patients frequently present with a variable-sized hyperpigmented ulcerative lesion that is commonly found on the head or neck and has no male or female predominance [3,7-9]. It appears to target the older population, affecting patients in the fifth and sixth decades of life [7-9]. The tumor tends to rapidly metastasize to the lymph nodes, which confers a poor prognosis [3].

The long growth course and varying presentations of SCACP complicate the diagnosis and management plan. Therefore, clinicians rely on tissue analysis and histopathological findings to establish a definitive diagnosis. Histopathology typically reveals multilayered epithelium with luminal decapitation and a lymphocytic infiltrate with associated hyperchromatic nuclei and mitotic figures within the stroma [7]. Deep papillary invaginations of keratinizing squamous epithelium and glandular elements span throughout the epidermis and dermis [10]. These invaginations are comprised of a two-layered epithelium, demonstrating columnar cells and cuboidal cells of the inner and outer layers, respectively [11]. SCACP is recognized by the presence of numerous atypical cystic structures with abundant mitoses within the dermis and subcutaneous tissues [11]. This case describes the rapid onset of SCACP arising de novo on the patient’s scalp. Based on the review of current literature, there are less than 30 cases of SCACP presenting on the scalp reported in the medical literature [11]. In contrast to other case studies describing a multi-step progression into SCACP, this case describes an aggressive malignant transformation into SCACP without any preceding signs.

## Case presentation

**Figure 1:**
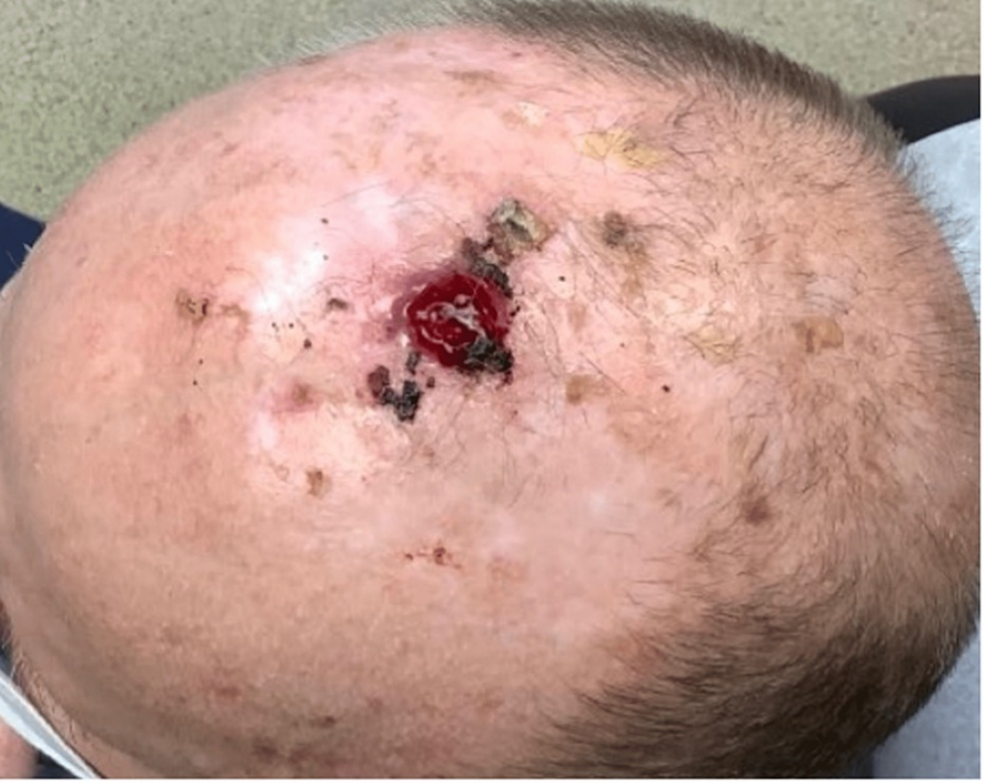
Ulcerating SCACP lesion on the mid-frontal scalp.

**Figure 2:**
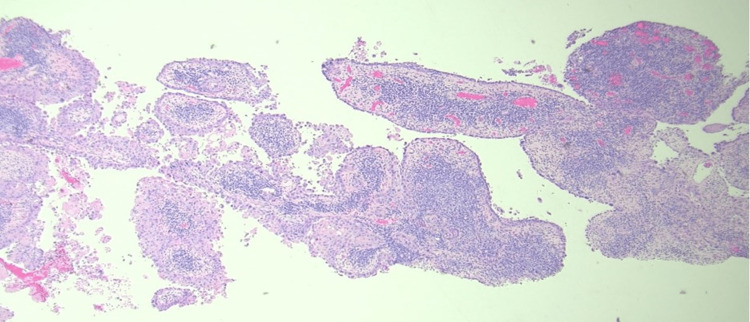
Higher magnification shows papillary architecture and prominent nuclear atypia (H&E. X40).

**Figure 3:**
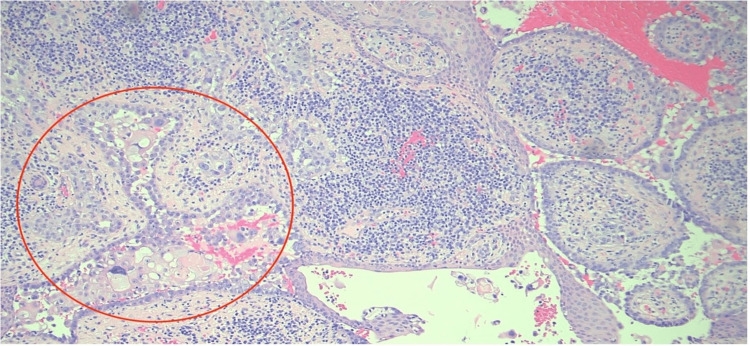
The neoplastic aggregates (red circle) in the dermis with dense inflammatory cell infiltrate (H&E. X100).

An 83-year-old white male with a past medical history of non-melanoma skin cancers presented to the clinic for an evaluation of a suspicious skin lesion located on the mid-frontal scalp (Figure 1). Upon gross examination, a 1.6-cm by 1.6-cm erythematous and tender nodule was seen with an associated hyperkeratotic scale. According to the patient, the lesion first appeared several months ago and had never been addressed before. The patient’s past medical history was significant for basal cell carcinoma, squamous cell carcinoma, and asteatosis cutis. Given such a strong predisposition to developing skin cancers, a shave biopsy was performed to rule out an invasive squamous cell carcinoma. Based on an independent review by a pathologist, histopathology revealed a malignant papillary neoplasm with features of SCACP (Figures 2, 3). Immunostaining further revealed strong positivity for p63 and CK5/6 markers, with focal reactivity for CK7 and CEA. The KI-67 tumor proliferation index was also considerably increased, but an absence of GCDFP-15 expression was noted. The diagnosis of an invasive SCACP was established. Mohs surgery was offered to the patient considering the anatomical location of the tumor. The lesion was excised in two consecutive stages, with the final excision measuring 3.2 cm by 2.8 cm. The patient was seen for a follow-up visit four days post-operation and had not yet presented with any new skin changes. 

## Discussion

One of the first cases of SCACP is dated as early as 1917, but there are fewer than 50 cases that have been reported thus far [11]. Being a cutaneous adenocarcinoma, the tumor typically originates from the apocrine glands in the presence of a long-standing benign lesion [4-6]. The etiology of the tumor remains unclear, but genetic predisposition, along with the positive history of HPV, seems to play a pivotal role in the carcinogenesis of the lesion [4,6]. Overactivation of the RAS-MAPK pathway seems to trigger aberrant growth of benign precursors of SCACP, including SCAP and NS [4]. In 40% of SCACP cases presenting in the head and neck region, the tumors have been commonly associated with the transformation of a pre-existing benign NS [7,8].

Skin presentation is non-specific and can be easily confused with other skin cancers, which makes this malignancy so perplexing in nature. The tumor appears as a flesh-colored, raised nodule that increases in size over time, followed by an abrupt ulcerating exudate with subsequent crust production [10]. The differential diagnosis primarily includes squamous cell carcinoma (SCC), basal cell carcinoma (BCC), cutaneous lymphoma, factitious dermatitis, and pyogenic granuloma, as these lesions grossly resemble the SCACP [4,12,13]. Furthermore, the tumor can be confused with papillary eccrine or tubular apocrine adenomas, as these benign growths stem from the same sweat gland precursor as SCACP [14]. Additionally, it is crucial to keep in mind cutaneous metastases from other tumors in the differential diagnosis. Cutaneous metastases are infamously known for imitating other diseases due to the wide range of their clinical presentations [15]. Moreover, the head and scalp are one of the most common sites of metastatic cutaneous malignancies originating from other visceral organs, including lung, stomach, pancreas, colon, ovary, and thyroid cancers [16-19].

Although there are no specific histological stains for SCACP, certain tumor markers have been strongly associated with tumor progression, including tumor protein p63 and gross cystic disease fluid protein (GCDFP)-15 [3-7]. Furthermore, the majority of SCACP specimens have been found to stain positively for periodic acid Schiff (PAS), cytokeratin (CK) 7 and (CK) 5/6, epithelial membrane antigen (EMA), and carcinoembryonic antigen (CEA) [3-5]. Among these tumor markers, positive expression of both p63 and CK-7 strongly suggests the diagnosis of SCACP [3,4]. Given its uncommon presentation, treatment modalities are not well established. Nonetheless, complete surgical excision of the lesion and close follow-up are the most reasonable management plans for patients with SCACP [12]. In some patients who are poor surgical candidates, chemotherapy and radiation may be offered [20]. Nonetheless, recurrences are still possible and have been reported in less than 20% of cases; however, there are currently no definitive markers to aid with establishing the prognosis for patients with a history of SCACP [1].

## Conclusions

SCACP is an exceptionally rare cutaneous adnexal tumor that is encountered by clinicians worldwide. The clinical presentation and morphology of the neoplasm can be easily confused with the variety of other skin cancers and cutaneous metastases. Thus, prompt biopsy along with histopathological evaluation is critical to the accurate diagnosis of SCACP. Particularly, observing the positive expression of p63 and CK5/6 markers, along with the reactivity towards CK7 and CEA stains, is strongly associated with the SCACP diagnosis. It is crucial to differentiate this tumor from other cutaneous neoplasms due to its rare presentation and aggressive lymph node metastases that have been previously reported in the literature.
